# *N*-Benzyl­pyridine-2-sulfonamide

**DOI:** 10.1107/S1600536809018054

**Published:** 2009-05-29

**Authors:** Xiao-Ping Chen, Shou-Fa Han

**Affiliations:** a Department of Chemistry and the Key Laboratory for Chemical Biology of Fujian Province, College of Chemistry and Chemical Engineering, Xiamen University, Xiamen 361005, People’s Republic of China

## Abstract

The title compound, C_12_H_12_N_2_O_2_S, was obtained by the reaction of 2-mercaptopyridine and benzyl­amine. The dihedral angle between the benzene and pyridine rings is 75.75 (9)°. In the crystal, mol­ecules are linked into chains along the *c* axis by N—H⋯O and N—H⋯N hydrogen bonds; the chains are cross-linked into a two-dimensional network parallel to the *bc* plane *via* C—H⋯O hydrogen bonds.

## Related literature

For the synthesis, see: Wright *et al.* (2006 ▶). For applications of sulfonamides, see: Connor (1998 ▶). For the structure of *N*-benzyl­quinoline-8-sulfonamide, see: Andrighetti-Fröhner *et al.* (2006 ▶).
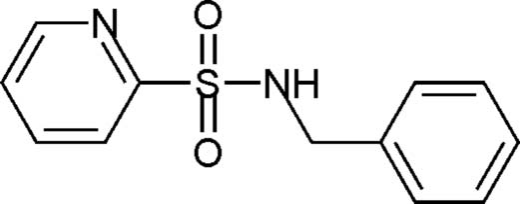


## Experimental

### 

#### Crystal data

C_12_H_12_N_2_O_2_S
*M* *_r_* = 248.30
Monoclinic, 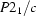

*a* = 11.099 (2) Å
*b* = 10.709 (2) Å
*c* = 9.513 (2) Å
β = 91.893 (4)°
*V* = 1130.1 (4) Å^3^
*Z* = 4
Mo *K*α radiation
μ = 0.28 mm^−1^
*T* = 173 K
0.50 × 0.20 × 0.18 mm

#### Data collection

Bruker SMART APEX area-detector diffractometer
Absorption correction: multi-scan (*SADABS*; Bruker, 2001 ▶) *T*_min_ = 0.823, *T*_max_ = 1.00 (expected range = 0.783–0.951)
5922 measured reflections
2195 independent reflections
2078 reflections with *I* > 2σ(*I*)
*R*_int_ = 0.021

#### Refinement

*R*[*F* ^2^ > 2σ(*F* ^2^)] = 0.038
*wR*(*F* ^2^) = 0.101
*S* = 1.00
2195 reflections
157 parameters
H atoms treated by a mixture of independent and constrained refinement
Δρ_max_ = 0.32 e Å^−3^
Δρ_min_ = −0.40 e Å^−3^

### 

Data collection: *SMART* (Bruker, 2001 ▶); cell refinement: *SAINT* (Bruker, 2001 ▶); data reduction: *SAINT*; program(s) used to solve structure: *SHELXS97* (Sheldrick, 2008 ▶); program(s) used to refine structure: *SHELXL97* (Sheldrick, 2008 ▶); molecular graphics: *ORTEP-3 for Windows* (Farrugia, 1997 ▶); software used to prepare material for publication: *SHELXL97*.

## Supplementary Material

Crystal structure: contains datablocks I, global. DOI: 10.1107/S1600536809018054/ci2800sup1.cif

Structure factors: contains datablocks I. DOI: 10.1107/S1600536809018054/ci2800Isup2.hkl

Additional supplementary materials:  crystallographic information; 3D view; checkCIF report

## Figures and Tables

**Table 1:** Hydrogen-bond geometry (Å, °)

*D*—H⋯*A*	*D*—H	H⋯*A*	*D*⋯*A*	*D*—H⋯*A*
N1—H1⋯N2^i^	0.82 (2)	2.49 (2)	3.264 (2)	157 (2)
N1—H1⋯O1^i^	0.82 (2)	2.50 (2)	3.111 (2)	132 (2)
C4—H4⋯O1^ii^	0.95	2.52	3.406 (2)	154
C5—H5⋯O2^iii^	0.95	2.51	3.121 (2)	122

Symmetry codes: (i) 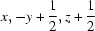; (ii) 
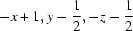
; (iii) 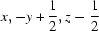.

**Fig. 1.**
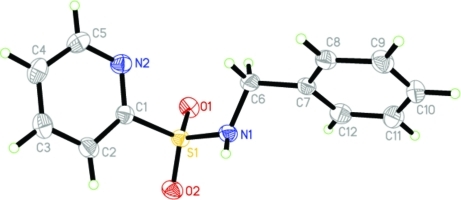
The molecular structure of the compound, with 50% probability displacement ellipsoids (arbitrary spheres for H atoms).

## supplementary crystallographic information

### Comment

Sulfonamides are an important category of pharmaceutical compounds with a broad spectrum of biological activities, as good antibacterials, diuretics, anticonvulsants, and HIV protease inhibitors (Connor, 1998).

The molecular structure of the title compound is shown in Fig. 1. Bond lengths and angles are comparable to those observed for *N*-benzylquinoline-8-sulfonamide (Andrighetti-Fröhner *et al.*, 2006). The C1—S1—N1—C6 torsion angle is -71.85 (15)°. The dihedral angle between the benzene and pyridine rings is 75.75 (9)°.

Hydrogen bonding plays a significant role in stabilizing the crystal structure; see Table 1 for geometric parameters and symmetry operations. The molecules are linked into a chain along the *c* axis by N—H···O and N—H···N hydrogen bonds. The chains are cross-linked *via* C—H···O hydrogen bonds to form a two-dimensional network parallel to the *bc* plane.

### Experimental

The title compound was synthesized using a similar synthetic method for the preparation of heteroaryl sulfonamides (Wright *et al.*, 2006). 2-Mercaptopyridine (0.56 g, 5 mmol) was stirred in a mixture of 25 mL of dichloromethane and 25 mL of 1 *M* HCl in a 125 ml flask for 8 min at 263 to 268 K. Cold sodium hypochlorite (6% solution, 0.68 *M*, 26 ml, 18 mmol, 3.3 equiv) was then added dropwise with very rapid stirring, maintaining the internal temperature at 263 to 268 K. The mixture was stirred for 30 min at 263 to 268 K after the addition was completed, the mixture was transferred to a separatory funnel (pre-cooled with ice water) and the dichloromethane layer was rapidly separated and collected in a clean 125 ml flask cooled in a ice-salt bath. Benzylamine (1.1 ml, 10 mmol) was added with stirring, when the dichloromethane layer became a white suspension, the flask was removed to an ice-water bath and the suspension was stirred for 30 min at 273 K. The suspension was then washed with 1 *M* HCl, then with water and brine. Drying (MgSO_4_) and concentration afforded the title compound as a white solid with 81% yield. Single crystals of the title compound were grown in a petroleum ether-ethyl acetate solution (3:1 *v*/*v*) by slow evaporation.

### Refinement

Atom H1 was located in a difference map and its positional parameters were refined. The remaining H atoms were positioned geometrically [C-H = 0.95 Å (aromatic) and 0.99 Å (methylene)] and were included in the refinement in the riding-model approximation. The isotropic displacement parameters were set at 1.2 times *U*_eq_ of the parent atoms.

### Crystal data

**Table:**

C_12_H_12_N_2_O_2_S	*F*(000) = 520
*M**_r_* = 248.30	*D*_x_ = 1.459 Mg m^−^^3^
Monoclinic, *P*2_1_/*c*	Mo *K*α radiation, λ = 0.71073 Å
Hall symbol: -P 2ybc	Cell parameters from 4484 reflections
*a* = 11.099 (2) Å	θ = 2.6–28.2°
*b* = 10.709 (2) Å	µ = 0.28 mm^−^^1^
*c* = 9.513 (2) Å	*T* = 173 K
β = 91.893 (4)°	Needle, colourless
*V* = 1130.1 (4) Å^3^	0.50 × 0.20 × 0.18 mm
*Z* = 4	

### Data collection

**Table:**

Bruker SMART APEX area-detector diffractometer	2195 independent reflections
Radiation source: fine-focus sealed tube	2078 reflections with *I* > 2σ(*I*)
graphite	*R*_int_ = 0.021
φ and ω scans	θ_max_ = 26.0°, θ_min_ = 1.8°
Absorption correction: multi-scan (*SADABS*; Bruker, 2001)	*h* = −12→13
*T*_min_ = 0.823, *T*_max_ = 1.00	*k* = −11→13
5922 measured reflections	*l* = −11→11

### Refinement

**Table:**

Refinement on *F*^2^	Primary atom site location: structure-invariant direct methods
Least-squares matrix: full	Secondary atom site location: difference Fourier map
*R*[*F*^2^ > 2σ(*F*^2^)] = 0.038	Hydrogen site location: inferred from neighbouring sites
*wR*(*F*^2^) = 0.101	H atoms treated by a mixture of independent and constrained refinement
*S* = 1.00	*w* = 1/[σ^2^(*F*_o_^2^) + (0.0567*P*)^2^ + 0.7208*P*] where *P* = (*F*_o_^2^ + 2*F*_c_^2^)/3
2195 reflections	(Δ/σ)_max_ = 0.001
157 parameters	Δρ_max_ = 0.32 e Å^−^^3^
0 restraints	Δρ_min_ = −0.40 e Å^−^^3^

### Special details

**Table:**

Geometry. All e.s.d.'s (except the e.s.d. in the dihedral angle between two l.s. planes) are estimated using the full covariance matrix. The cell e.s.d.'s are taken into account individually in the estimation of e.s.d.'s in distances, angles and torsion angles; correlations between e.s.d.'s in cell parameters are only used when they are defined by crystal symmetry. An approximate (isotropic) treatment of cell e.s.d.'s is used for estimating e.s.d.'s involving l.s. planes.
Refinement. Refinement of *F*^2^ against ALL reflections. The weighted *R*-factor *wR* and goodness of fit *S* are based on *F*^2^, conventional *R*-factors *R* are based on *F*, with *F* set to zero for negative *F*^2^. The threshold expression of *F*^2^ > σ(*F*^2^) is used only for calculating *R*-factors(gt) *etc*. and is not relevant to the choice of reflections for refinement. *R*-factors based on *F*^2^ are statistically about twice as large as those based on *F*, and *R*- factors based on ALL data will be even larger.

### Fractional atomic coordinates and isotropic or equivalent isotropic displacement parameters (Å^2^)

**Table:**

	*x*	*y*	*z*	*U*_iso_*/*U*_eq_	
S1	0.29480 (4)	0.35956 (4)	−0.07138 (4)	0.02300 (15)	
O1	0.23235 (11)	0.37287 (12)	−0.20382 (13)	0.0297 (3)	
O2	0.35175 (11)	0.46543 (11)	−0.00687 (13)	0.0303 (3)	
N1	0.20157 (13)	0.30466 (14)	0.03864 (16)	0.0247 (3)	
H1	0.2331 (19)	0.299 (2)	0.118 (2)	0.030*	
N2	0.37751 (14)	0.15428 (14)	−0.18353 (16)	0.0288 (3)	
C1	0.40853 (15)	0.24506 (16)	−0.09461 (17)	0.0242 (4)	
C2	0.51786 (16)	0.25414 (18)	−0.02210 (19)	0.0296 (4)	
H2	0.5353	0.3219	0.0397	0.036*	
C3	0.60085 (17)	0.16046 (19)	−0.0433 (2)	0.0336 (4)	
H3	0.6775	0.1622	0.0041	0.040*	
C4	0.57087 (17)	0.06477 (18)	−0.13383 (19)	0.0332 (4)	
H4	0.6264	−0.0009	−0.1497	0.040*	
C5	0.45909 (17)	0.06519 (18)	−0.20149 (19)	0.0329 (4)	
H5	0.4393	−0.0014	−0.2640	0.039*	
C6	0.11909 (16)	0.20325 (17)	−0.00404 (19)	0.0297 (4)	
H6A	0.1627	0.1226	0.0006	0.036*	
H6B	0.0903	0.2166	−0.1025	0.036*	
C7	0.01310 (15)	0.19830 (16)	0.09002 (17)	0.0240 (4)	
C8	−0.01617 (16)	0.08823 (16)	0.15529 (19)	0.0274 (4)	
H8	0.0322	0.0162	0.1424	0.033*	
C9	−0.11535 (16)	0.08132 (18)	0.23949 (19)	0.0321 (4)	
H9	−0.1354	0.0046	0.2830	0.039*	
C10	−0.18463 (17)	0.18527 (19)	0.2601 (2)	0.0345 (4)	
H10	−0.2523	0.1809	0.3185	0.041*	
C11	−0.15598 (17)	0.29609 (18)	0.1958 (2)	0.0353 (4)	
H11	−0.2037	0.3683	0.2104	0.042*	
C12	−0.05840 (17)	0.30255 (17)	0.1105 (2)	0.0301 (4)	
H12	−0.0399	0.3790	0.0653	0.036*	

### Atomic displacement parameters (Å^2^)

**Table:**

	*U*^11^	*U*^22^	*U*^33^	*U*^12^	*U*^13^	*U*^23^
S1	0.0240 (2)	0.0224 (2)	0.0226 (2)	−0.00314 (15)	0.00148 (16)	0.00164 (15)
O1	0.0300 (6)	0.0336 (7)	0.0254 (7)	−0.0023 (5)	−0.0006 (5)	0.0055 (5)
O2	0.0312 (6)	0.0233 (6)	0.0364 (7)	−0.0054 (5)	−0.0001 (5)	0.0000 (5)
N1	0.0253 (7)	0.0274 (8)	0.0215 (7)	−0.0047 (6)	0.0020 (6)	−0.0024 (6)
N2	0.0323 (8)	0.0296 (8)	0.0245 (8)	−0.0012 (6)	0.0005 (6)	−0.0012 (6)
C1	0.0248 (8)	0.0264 (8)	0.0215 (8)	−0.0040 (7)	0.0046 (6)	0.0024 (6)
C2	0.0260 (9)	0.0326 (9)	0.0303 (9)	−0.0045 (7)	0.0012 (7)	0.0000 (7)
C3	0.0259 (9)	0.0405 (10)	0.0346 (10)	−0.0009 (8)	0.0040 (7)	0.0058 (8)
C4	0.0355 (10)	0.0352 (10)	0.0295 (9)	0.0068 (8)	0.0098 (7)	0.0040 (8)
C5	0.0415 (10)	0.0307 (9)	0.0266 (9)	0.0020 (8)	0.0039 (8)	−0.0030 (7)
C6	0.0315 (9)	0.0287 (9)	0.0293 (9)	−0.0085 (7)	0.0084 (7)	−0.0066 (7)
C7	0.0233 (8)	0.0270 (8)	0.0217 (8)	−0.0053 (7)	0.0000 (6)	−0.0029 (6)
C8	0.0276 (8)	0.0240 (8)	0.0306 (9)	−0.0019 (7)	0.0003 (7)	−0.0022 (7)
C9	0.0340 (9)	0.0310 (9)	0.0315 (9)	−0.0079 (8)	0.0042 (7)	0.0035 (8)
C10	0.0275 (9)	0.0421 (11)	0.0343 (10)	−0.0065 (8)	0.0088 (7)	−0.0059 (8)
C11	0.0292 (9)	0.0331 (10)	0.0438 (11)	0.0038 (8)	0.0020 (8)	−0.0049 (8)
C12	0.0329 (9)	0.0251 (9)	0.0323 (9)	−0.0015 (7)	−0.0008 (7)	0.0022 (7)

### Geometric parameters (Å, °)

**Table:**

S1—O1	1.4249 (13)	C5—H5	0.95
S1—O2	1.4268 (13)	C6—C7	1.502 (2)
S1—N1	1.6073 (15)	C6—H6A	0.99
S1—C1	1.7787 (18)	C6—H6B	0.99
N1—C6	1.469 (2)	C7—C8	1.376 (2)
N1—H1	0.82 (2)	C7—C12	1.387 (3)
N2—C1	1.327 (2)	C8—C9	1.385 (3)
N2—C5	1.330 (2)	C8—H8	0.95
C1—C2	1.379 (2)	C9—C10	1.371 (3)
C2—C3	1.381 (3)	C9—H9	0.95
C2—H2	0.95	C10—C11	1.377 (3)
C3—C4	1.373 (3)	C10—H10	0.95
C3—H3	0.95	C11—C12	1.376 (3)
C4—C5	1.379 (3)	C11—H11	0.95
C4—H4	0.95	C12—H12	0.95
			
O1—S1—O2	119.76 (8)	N1—C6—C7	110.77 (14)
O1—S1—N1	107.91 (8)	N1—C6—H6A	109.5
O2—S1—N1	107.23 (8)	C7—C6—H6A	109.5
O1—S1—C1	106.59 (8)	N1—C6—H6B	109.5
O2—S1—C1	107.16 (8)	C7—C6—H6B	109.5
N1—S1—C1	107.67 (8)	H6A—C6—H6B	108.1
C6—N1—S1	119.95 (12)	C8—C7—C12	118.78 (16)
C6—N1—H1	116.0 (15)	C8—C7—C6	120.01 (16)
S1—N1—H1	111.2 (15)	C12—C7—C6	121.20 (16)
C1—N2—C5	116.37 (15)	C7—C8—C9	120.72 (17)
N2—C1—C2	125.17 (17)	C7—C8—H8	119.6
N2—C1—S1	114.46 (13)	C9—C8—H8	119.6
C2—C1—S1	120.36 (14)	C10—C9—C8	119.98 (17)
C1—C2—C3	117.12 (17)	C10—C9—H9	120.0
C1—C2—H2	121.4	C8—C9—H9	120.0
C3—C2—H2	121.4	C9—C10—C11	119.85 (18)
C4—C3—C2	119.00 (18)	C9—C10—H10	120.1
C4—C3—H3	120.5	C11—C10—H10	120.1
C2—C3—H3	120.5	C12—C11—C10	120.18 (18)
C3—C4—C5	119.10 (18)	C12—C11—H11	119.9
C3—C4—H4	120.5	C10—C11—H11	119.9
C5—C4—H4	120.5	C11—C12—C7	120.48 (17)
N2—C5—C4	123.24 (17)	C11—C12—H12	119.8
N2—C5—H5	118.4	C7—C12—H12	119.8
C4—C5—H5	118.4		
			
O1—S1—N1—C6	42.85 (16)	C2—C3—C4—C5	0.3 (3)
O2—S1—N1—C6	173.12 (13)	C1—N2—C5—C4	−0.4 (3)
C1—S1—N1—C6	−71.85 (15)	C3—C4—C5—N2	−0.1 (3)
C5—N2—C1—C2	0.8 (3)	S1—N1—C6—C7	−159.20 (13)
C5—N2—C1—S1	−178.54 (13)	N1—C6—C7—C8	−126.83 (17)
O1—S1—C1—N2	−34.71 (14)	N1—C6—C7—C12	54.6 (2)
O2—S1—C1—N2	−164.06 (12)	C12—C7—C8—C9	0.2 (3)
N1—S1—C1—N2	80.87 (14)	C6—C7—C8—C9	−178.38 (16)
O1—S1—C1—C2	145.94 (14)	C7—C8—C9—C10	−0.9 (3)
O2—S1—C1—C2	16.58 (16)	C8—C9—C10—C11	0.6 (3)
N1—S1—C1—C2	−98.49 (15)	C9—C10—C11—C12	0.3 (3)
N2—C1—C2—C3	−0.6 (3)	C10—C11—C12—C7	−1.0 (3)
S1—C1—C2—C3	178.71 (13)	C8—C7—C12—C11	0.7 (3)
C1—C2—C3—C4	0.0 (3)	C6—C7—C12—C11	179.29 (17)

### Hydrogen-bond geometry (Å, °)

**Table:**

*D*—H···*A*	*D*—H	H···*A*	*D*···*A*	*D*—H···*A*
N1—H1···N2^i^	0.82 (2)	2.49 (2)	3.264 (2)	157 (2)
N1—H1···O1^i^	0.82 (2)	2.50 (2)	3.111 (2)	132 (2)
C4—H4···O1^ii^	0.95	2.52	3.406 (2)	154
C5—H5···O2^iii^	0.95	2.51	3.121 (2)	122

Symmetry codes: (i) *x*, −*y*+1/2, *z*+1/2; (ii) −*x*+1, *y*−1/2, −*z*−1/2; (iii) *x*, −*y*+1/2, *z*−1/2.

## References

[bb1] Andrighetti-Fröhner, C. R., da Silva, L. E., Nunes, R. J., Simões, C. M. O. & Foro, S. (2006). *Acta Cryst.* E**62**, o3693–o3694.

[bb2] Bruker (2001). *SAINT*, *SMART* and *SADABS* Bruker AXS Inc., Madison, Wisconsin, USA.

[bb3] Connor, E. E. (1998). *Prim. Care Update Ob. Gyn.* **5**, 32–35.

[bb4] Farrugia, L. J. (1997). *J. Appl. Cryst.* **30**, 565.

[bb5] Sheldrick, G. M. (2008). *Acta Cryst.* A**64**, 112–122. 10.1107/S0108767307043930 18156677

[bb6] Wright, S. W. & Hallstrom, K. N. (2006). *J. Org. Chem.* **71**, 1080–1084. 10.1021/jo052164+ 16438524

